# Fortunate molecules boost signal to background ratio and localization precision in correlation based single molecule localization microscopy

**DOI:** 10.1038/s42003-024-07153-x

**Published:** 2024-12-23

**Authors:** Aravinth S, Francesca Cella Zanacchi, Partha Pratim Mondal

**Affiliations:** 1 Department of Instrumentation and Applied Physics, Indian Institute of Science, Bangalore, 560012 India; 2 Department of Physics, University of Pisa, Pisa, Italy; 3 Center for Instrument Sharing of the University of Pisa (CISUP), Pisa, Italy; 4 Centre for Cryogenic Technology, Indian Institute of Science, Bangalore, 560012 India

**Keywords:** Super-resolution microscopy, Fluorescence imaging, Wide-field fluorescence microscopy

## Abstract

Single-molecule localization microscopy (SMLM) can decipher fine details that are otherwise impossible using diffraction-limited microscopy. Often, the reconstructed super-resolved images suffer from noise, strong background and are prone to false detections that may impact quantitative imaging. To overcome these limitations, we propose a technique (*corrSMLM*) that recognizes and detects fortunate molecules (molecules with long blinking cycles) from the recorded data. The method uses correlation between two or more consecutive frames to identify and isolate fortunate molecules that blink longer than the standard blinking period of a molecule. The *corrSMLM* is based on the fact that random fluctuations (noise) do not last longer (usually limited to a single frame). In contrast, fortunate molecules consistently fluoresce for extended periods and hence appear on more than one frame. Accordingly, strongly correlated spots (representing fortunate molecules) are compared in the consecutive frames, followed by data integration to determine their position and localization precision. The technique addresses two significant problems that plague existing SMLM : (1) false detection due to random noise that contributes to a strong background and (2) poor localization leading to overall low resolution. To demonstrate, *corrSMLM* is used for imaging fixed NIH3T3 cells (transfected with Dendra2-Actin, Dendra2-Tubulin, and mEos-Tom20 plasmid DNA). The super-resolved images show a significant reduction in background noise ( > 1.5 fold boost in SBR) and  > 2-fold improvement in localization precision as compared to standard SMLM. Intensity analysis based on the number of molecules suggests that corrSMLM better corroborates the raw data and preserves finer features (e.g., edges), which are wiped out in standard SMLM. Overall, an improvement is noted in the localization precision and spatial resolution. The proposed technique is anticipated to advance SMLM and is expected to contribute to a better understanding of single-molecule dynamics in a cellular environment.

## Abstract (web summary)

Detection of fortunate molecules by correlation-based single-molecule localization microscopy enhances signal-to-background ratio and improves spatial resolution, leading to better resolved single-molecule ensembles in a cellular system.

## Introduction

In biology, molecules often assemble and arrange in a specific pattern to perform biological functions. Visualizing these patterns and understanding their arrangement is of utmost importance and has ramifications ranging from cell to disease biology. In this respect, single molecule localization microscopy (SMLM) and related microscopy techniques have proven to be valuable tools for unraveling the nanoscopic world of biomolecular complexes.^1–5^.

Since the classical work of Moerner in the year 1989 and inception of super-resolution microscopy in the subsequent years with the advent of STED, SI and SMLM techniques (PALM, fPALM and STORM), super-resolution imaging has taken a giant step towards unveiling many biological processes occurring at single molecule level^1,6–12^. Over the years, a family of powerful techniques have emerged, enabling detailed study of molecular complexes in cells and organs. Recent advances in SMLM such as, POSSIBLE, STED, MINFLUX, dSTORM, SIMPLE and ROSE have shown sub-10 nm resolution and better quantitative tool^3,13–21^. In this regard, the integration of light sheet and super-resolution have shown great promise^22^. Specifically, the last decade has seen many variants including, ground-state depletion microscopy (GSDIM)^23^, super-resolution optical fluctuation imaging (SOFI)^24^, points accumulation for imaging in nanoscale topography (PAINT)^25,26^, simultaneous multiplane imaging-based localization encoded (SMILE)^27,28^, individual molecule localization-selective plane illumination microscopy (IML-SPIM)^22^, MINFLUX^18^,POSSIBLE microscopy, scanning SMLM^29^ and others^30–37^. These techniques have advanced our understanding of biological processes in cellular system. For example, SMLM has revealed the nanoscopic world of macromolecular complexes and its functioning in a cellular environment including, apoptopic pores^38^, nuclear pore complex^39^, endocytic machinery^40^, cytoskeletal structure^41^, cellular adhesion^42^, HA clustering^2,29,43–45^ and many more. LIVE-PAINT has demonstrated imaging a number of different peptide binding protein pairs (TRAP4-MEEVF and SYNZIP18-SYNZIP17) inside live S. cerevisiae^46^. IML-SPIM has enabled super-resolution imaging of human mammary MCF10A cell spheroids expressing H2B-PAmCherry^22^. A recent technique based on fast sCMOS technology (termed as, temporally-resolved SMLM) enabled visualization of dynamic HA cluster formation in a live cell^47^. Multicolor variant such as multicolor STORM facilitated imaging of large volumes with ultrathin sectioning of ganglion cells to understand the nanoscale co-organization of AMPA receptors and their spatial correlation^48–50^. A recent technique primarily based on RESOLFT microscopy have shown 3D imaging of single molecule organization in a cell^51^. The technique has reported volumetric imaging of exosomes labelled with CD63-rsEGFP2 in live U2OS cells. Other techniques predominately based on evanescent light such as SMILE has demonstrated volume imaging capabilities in a single cell^28^.

The last few years have seen integration of SPIM techniques with other imaging techniques for enabling studies which are earlier thought out of reach. IML-SPIM has enabled super-resolution imaging at large field-of-view (FOV). Multiphoton variant of super-resolution microscopy has enabled better penetration depths suitable for scattering medium such as tissue^52,53^. SMILE technique combines the strengths of SMLM and TIRF, facilitating 3D imaging of viral (Hemaglutinin) transfected NIH3T3 cells^27,28^. Recently, DNA-PAINT and fluorescence lifetime PAINT (FL-PAINT) has demonstrated the capability to image multiple targets simultaneously in a HeLa cell^54^. Bessel beam STED has shown deep penetration capability that can be used for tissue / organ imaging^55^. In another application, SMLM is realized using a Bessel beam for 3D imaging of nucleoporin Nup153 expressed in HeLa cell nucleus^56^. Recently developed MINFLUX and MINSTED techniques which is a combination of SMLM and STED has demonstrated imaging of nuclear-pore complex with high spatio-temporal resolution^13,17,57^. SMLM techniques are in their early stages and the emerging potential variants are expected to reveal fundamental aspects of biological processes in the coming decade.

However, SMLM techniques suffer from strong background, and poor localization precision. This is primarily due to laser intensity fluctuations, detector limitations (read noise, quantum efficiency etc.) and sub-optimal sample preparation. The background noise mentioned here is basically the noise present in the raw data, the origin of which could be fluorescence background noise, electronic noise, thermal noise, and other noises that give rise to false detections. Irrespective of the single molecule detection algorithm these noises are also detected as molecules and contribute to the reconstructed image, affecting both the image resolution and further quantitative analysis. Ideally, single molecule imaging would greatly benefit if the background noise could be suppressed. Towards this goal, a new technique (corrSMLM) is proposed that uses a correlation method to merge single molecules that remain bright for more than one image/frame (also termed as, fortunate molecules), and reject any other bright spot that is inconsistent. The technique eliminates random noise because the chance of getting the noise in the same pixel location for consecutive frames is rare. Therefore techniques that can overcome background noise are highly beneficial and are expected to expand the reach of super-resolution technique beyond the capabilities of standard super-resolution microscopy. In this report, we propose a correlation based SMLM (*corrSMLM*) technique that uses correlation of single molecule events to identify fortunate molecules (bright molecules with large emission cycles) from consecutive frames of the recorded data. The information (such as, emitted photons from correlated single molecules, degree of correlation and number of correlated frames) are then integrated to determine localization precision, position of the single molecule and related parameters. Results show a multi-fold improvement in the localization precision, and high signal-to-background ratio (SBR) which facilitates imaging close to sub-10nm regime.

## Results

The immense potential of single molecule based super-resolution microscopy can be harnessed only if the quality of reconstructed image improves substantially. Among others, localization precision and signal-to-background ratio (SBR) are key factors that are essential for high quality imaging, and forms the basis for studying biological processes in great detail. This calls for methods that are promising and does not require substantial change in system hardware. *corrSMLM* is one such technique that can be readily integrated with most of the existing family of super-resolution microscopy techniques.

**Fig. 1:**
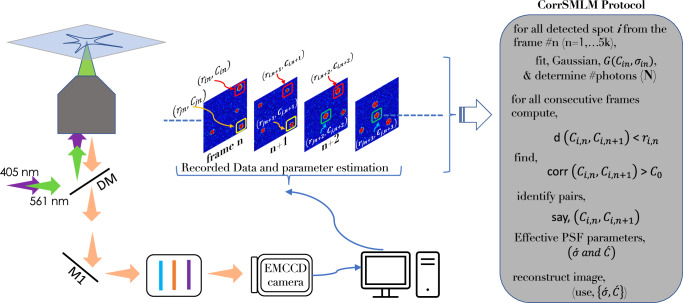
Correlation based Single Molecule Localization Microscopy (*corrSMLM*). A graphical representation of *corrSMLM* and related key steps (optical and computational). The steps involve, spot detection, pair identification, correlation, Gaussian fitting and parameters estimation (localization precision, position and number of emitted photons), and finally, reconstruction of *corrSMLM* image. Detailed description of the reconstruction can be found in Supplementary Note 1.

The schematic diagram of the *corrSMLM* technique / method is shown in Fig. 1. The optical system is similar to that of a standard SMLM microscopy. Several single molecule images are recorded and processed to isolate the single molecules (bright spots) which are then fitted using a 2D Gaussian function ($G=A\,\exp [{(x-{x}_{0})}^{2}/2{\sigma }^{2}+{(y-{y}_{0})}^{2}/2{\sigma }^{2}]$) where, (*x*_0_, *y*_0_) and (*σ*) are the mean and standard deviation, respectively. This is followed by determining the position (centroid) and the number of photons. The centroid of extracted single molecules in each frame (say, *n*) are then compared with the single-molecule signatures in the preceding (frame *#n* − 1) and next frame (i.e, frame *#n* + 1). If the centroids are found to lie within the theoretically estimated diffraction-limited spot (*r* ~ 1.2*λ*/2*NA*), then the respective Gaussians are correlated. Depending upon the degree of correlation, the single molecule in the consecutive frames are identified as pair and sorted as shown in the adjoining table (see, Fig. 1 and Supplementary Note 1). Subsequently, the parameters are computed for the paired molecules. Since fortunate molecule emit for longer duration i.e, detectable in more than one consecutive frames they can be identified as a single molecule^58^. These elusive molecules has a PArticle Resolution shift (PAR-shift) towards single molecule limit, and thus have a better localization^47,58^. The other benefit is the elimination of false detections (such as, background noise) which pops-in during experimentation (detector read noise, intensity fluctuations, thermal noise and background). Finally, the informations related to position and total number of detected photons are combined for reconstructing the single molecule map / super-resolved image.

**Fig. 2:**
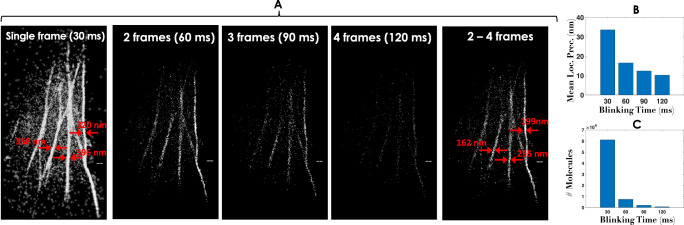
Correlation study for Dendra2-Actin transfected cells. **A** Single molecule data collection at 30*Hz* for Dendra2-Actin transfected cells, and consecutive frames are correlated to recognize fortunate molecules (single molecules that emit longer than average blinking period). Correlation (*χ* = 0.7) was carried out for 2, 3, and 4 frames that span an exposure time of 60, 90, 120*ms*. An additional image is reconstructed from all the single molecules collected in 2-4 consecutive frames. The width of Actin bundles are indicated by red arrows. **B**, **C** Corresponding statistics for mean localization precision and the number of molecules with blinking time are also shown. Reconstructed super-resolved images for, *χ* = 0.8, 0.9 can be found in Supplementary Note 2. The raw data is shown in Supplementary Video 1. Additional Dendra2-Actin data and FRC analsysis are presented in Supplementary Note 9. Scale bar = 1*μm*.

The reconstructed super-resolved images (both SMLM and *corrSMLM*) of Dendra2-Actin transfected cells for correlation value, *χ* = 0.7 are shown in Fig. 2. It becomes immediately evident that, correlated images are free from background noise, and there is a marked improvement in single molecule localization (decrease in average localization precision as shown in Fig. 2B). This is possible due to the detection of fortunate molecules that have longer blinking period. The effect is clearly evident from the resolved actin bundles, and suppression of background noise. Specifically, the widths (FWHM) of the actin bundles in the reconstructed images are also mentioned in Fig. 2A (see red arrows) that showed an improved localization performance. Another important aspect of single molecule imaging is the number of molecule that contribute to the final super-resolved image. The down-side of *corrSMLM* is probably the small number of fortunate molecules that represent the final image. Figure 2C clearly indicates that there is a decrease in fortunate molecules that blink for longer time (*τ* ≥ 60*ms*). This of-course reduces background and improves single molecule localization, but substantially reduces the number statistics (the number of single molecules) required for constructing an acceptable super-resolved image. This can easily be compensated by recording large data. Note that, the choice of exposure time depends on the ON-time (*T*_*on*_) of the molecules. This also decides the number of molecules that appears on consecutive frames. The histogram of number of molecules with respect to *T*_*on*_ is discussed in Supplementary Note 11.

**Fig. 3:**
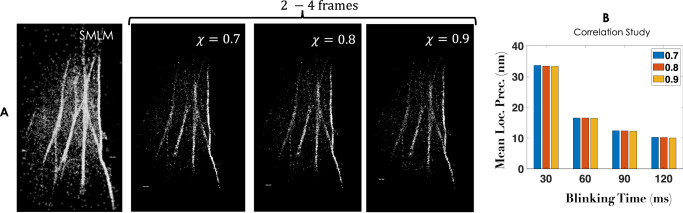
SMLM and *corrSMLM* reconstructed images (integrating all single molecules in 2-4 frames). **A** Images at varying correlation values, *χ* = 0.7, 0.8, 0.9. **B** Alongside localization precision plot is also displayed. A video comparing SMLM and *corrSMLM* is shown in Supplementary Video 2, and reconstructed images for varying blinking times are shown in Supplementary Note 2. Scale bar = 1*μm*.

The other aspect of *corrSMLM* is the degree of correlation with which two spots can be recognized as a single-molecule (referred as pair-molecule/fortunate molecule).Specifically, three different values of correlation factor, (*χ*) of ≥0.7, ≥0.8 and ≥0.9 are chosen and corresponding images are reconstructed as shown in Fig. 3A. It may be noted that, all the single molecules that blink for *τ*≥60*ms* (frames, 2-4) are combined to reconstruct super-resolution map. Alongside standard SMLM image is also shown for comparison. Although the number of molecules for reconstructed image with *χ* > 0.9 is less than *χ* > 0.7 and *χ* > 0.8, key features are well preserved. The corresponding mean localization precision for all *χ*-values are also plotted in Fig. 3B. This consistently show better localization of fortunate single molecules with localization precision  < 10*nm*. In addition, we noted that localization precision is not strongly dependent on the degree of correlation. This overall suggests the ability of *corrSMLM* to accurately recognize single molecules with long blinking period.

**Fig. 4:**
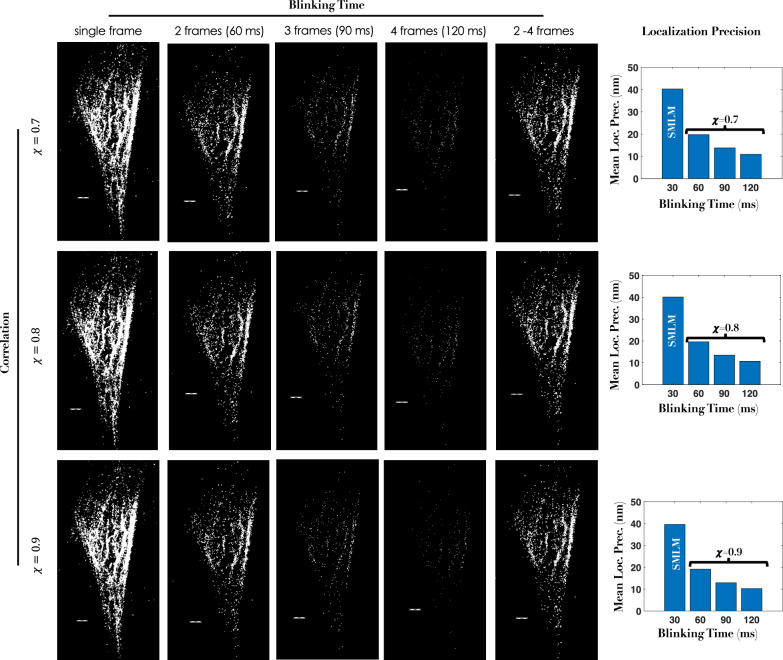
*corrSMLM* reconstructed images (tubulin structure labeled with Dendra2-tubulin) for different and correlation values (*χ* = 0.7 to 0.9), and varying blinking times (corresponding to molecules that are detected in 2, 3, 4 frames and for all the frames (2-4). Alongside localization precisions are also shown. The corresponding raw data along with super-resolved images (comparing SMLM and *corrSMLM*) is shown in Supplementary Videos 3 and 4. Scale bar = 1*μm*.

To strengthen and validate our technique, we considered another specimen where the cells were transfected with Dendra2-Tubulin plasmid DNA. A protocol similar to that of first sample is followed and the cell is fixed for imaging. Figure 4 shows reconstructed images at varying blinking times / number of frames and correlation factors, along with the localization precision. We observed a similar trend to that of first sample. It is clear that high-resolution images can be reconstructed using all fortunate molecules that blink for *τ* ≥ 60*ms* and appear in 2-4 frames. Alongside, mean localization precision with *#frames* / blinking time is also shown. It is evident that *corrSMLM* has the advantage of better number statistics, higher localization and an overall better SBR when compared to standard SMLM.

**Fig. 5:**
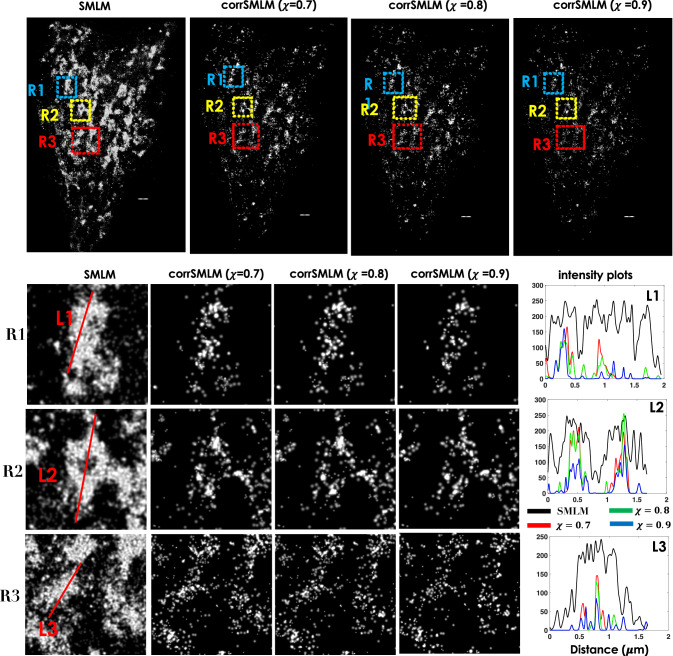
*corrSMLM* reconstructed super-resolved images of mEos-Tom20 transfected NIH3T3 cells (targeted to mitochondrial network). The super-resolved images for SMLM and *corrSMLM* with correlation values (*χ* = 0.7 to 0.9) considering all (2-4) frames. Alongside intensity plots are also shown that demonstrate elimination of background (noise) molecules. Region-wise (R1, R2, R3) comparison of SMLM and *corrSMLM* reconstructed images and video are shown in Supplementary Note 3 and Supplementary Video 7. The raw data along with reconstructed images can be found in Supplementary Videos 5 and 6. Scale bar = 1*μm*.

In another experiment, NIH3T3 cells were transfected with mEos-Tom20 plasmid DNA following similar protocol (see, materials and methods section). Figure 5 shows the super-resolved images reconstructed using SMLM and corrSMLM (for *χ* = 0.7, 0.8, 0.9). Three enlarged sections (marked by, A, B, C) are chosen to visually compare corrSMLM and SMLM. It is quite evident that mitochondrial network is better resolved which is predominantly due to the reduction of background molecules representing false detection. Here, signal is represented by fortunate molecules (that appear in ≥2 frames). As a result, correlation between subsequent frames help recognise consistent emission from fortunate molecules. Specifically, features are better visible in corrSMLM reconstructed images as compared to SMLM (see, enlarged regions (R1, R2, R3) and intensity plots in Fig. 5). This indicate feature-preserving nature of *corrSMLM*.

**Fig. 6:**
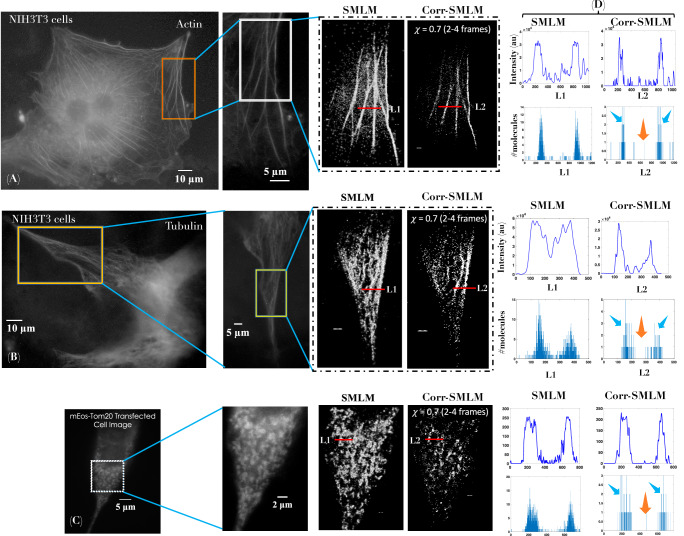
Comparison of *corrSMLM* and standard SMLM reconstructed images. **A**, **B**, **C** The widefield fluorescence image of Dendra2-Actin, Dendra2-Tubulin and mEos-Tom20 transfected cells. **D** The corresponding intensity plots along lines *L*1 and *L*2 passing through actin and tubulin structures are shown. Another plot shows the number of molecules representing fine structures (blue arrow) and background (orange arrow) is also displayed. Scale bar = 1*μm*.

**Fig. 7:**
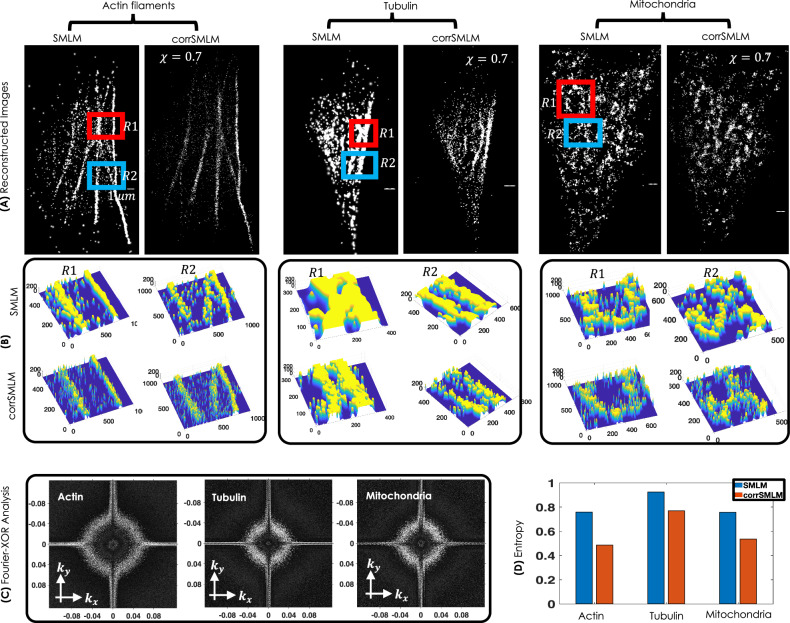
3D Visualization and statistical analysis. **A** Comparison of SMLM and *corrSMLM* for three different organelles (Actin filaments, Tubulin and Mitochondria) for similar number of counts. **B** 3D surface plot of few enlarged sections are also shown. **C** Bitwise Fourier analysis of reconstructed sub-cellular structures (Actin, Tubulin and Mitochondrial network). **D** Entropy measure (related to randomness) for corrSMLm and SMLM to determine order in the structures and quantify noise.

To accentuate the advantages of *corrSMLM* over SMLM, a separate comparison is carried out in Fig. 6, where, (A,B) shows widefield fluorescence images of Dendra2-Actin, Dendra2-Tubulin and mEos-Tom20 transfected cells. The corresponding region-of-interest is marked (red and yellow boxes). Alongside, standard SMLM and *corrSMLM*(*χ* = 0.7) (combined, 2-4 frames) super-resolved images for the ROIs are also shown. It is evident that, *corrSMLM* images are far superior than standard SMLM in terms of background noise. To determine the resolving power of *corrSMLM*, we have carried out line intensity plots across actin-filaments which shows a reduction in the FWHM of actin-filaments by a factor of  ~2. The corresponding localization precision is discussed in Supplementary Note 4. Moreover, this suggests an increase in the resolution of *corrSMLM* reconstructed images, which is also indicated in FRC analysis (see, Supplementary Note 6). In addition, we have counted the number of molecules that represent the actual structure (such as, actin filaments) and background (molecules in between the filaments). It is evident from the signal-to-background ratio (measured in terms of number of molecules) has improved substantially when compared to standard SMLM (see orange arrows in Fig. 6D). To ameliorate the effect of molecule statistics, we have carried out a comparison of *corrSMLM* and SMLM for similar number of single molecules as shown in Fig. 7. Reconstructed images along with enlarged 3D surface plots and intensity plots suggests a better background rejection for *corrSMLM* as compared to SMLM. In addition, frequency analysis is carried out to determine the presence of high frequencies that represents edge information and/or minute features in the image. We used bitwise XOR operation between corrSMLM and SMLM reconstructed images of intra-cellular structures (Actin, Tubulin and Mitochondria) as shown in Fig. 7C. XOR nullifies the low-frequency components present in the Fourier domain and retains only the desired high frequency components. Specifically, the ring represents frequencies corresponding to fine structures. The corresponding Fourier transformed images for Dendra2-Actin, Dendra2- Tubulin and mEos-Tom20 transfected cells is shown in Fig. S7-1 (Supplementary Note 7). The corresponding entropy bar plot is shown in Fig. 7D. This metric captures the randomness in super-resolved images. A high-level of order is apparent in *corrSMLM* as compared to standard SMLM, suggesting preservation of genuine fine features. Overall, the proposed *corrSMLM* technique is promising for super-resolution imaging that are prone to high noise and strong background with inbuilt feature preserving nature (better resolution).

All the findings related to *corrSMLM* are incorporated in tabular form as shown in Table 1. The performance of *corrSMLM* depends on few key factors such as, correlation factor, the number of correlated frames and the total number of fortunate molecules. These parameters determine the position, *#*photons per molecule, localization precision, and signal to background noise. It is apparent from table I that, localization of single molecules have improved by a factor of  ~2 i.e, mean localization precision is nearly-halved as compared to standard SMLM. This is closely linked to the number of photons emitted by single molecules. On the other hand, the signal-to-background ratio (measured in terms of *#* molecules) increased by nearly 1.5 times. Also the number of molecules necessary to represent an organelle structure (here, actin filament and tubulin network) have decreased by an order. This is predominantly due to the fact that number of molecules representing background/noise/false detections have reduced for *corrSMLM*. Overall, the benefits offered by *corrSMLM* over standard SMLM are note-worthy and beneficial for single molecule imaging techniques.

**Table 1:** Estimation of critical biophysical parameters (mean localization precision, signal-to-background ratio, number of emitted photons per molecule and total number of molecules) for *SMLM* and *corrSMLM*

Technique	organelle	SMLM		corrSMLM	
			0.7	0.8	0.9
Mean Loc. Prec.	Actin	33	15.3	15.3	15.2
(nm)	Tubulin	31	17.8	17.6	17.4
	Mitochondria	33	20.9	20.3	19.4
SBR	Actin	2.44	3.14	4.03	3.83
	Tubulin	3.4	3.4	4.2	4.5
	Mitochondria	2.46	4.57	4.8	4.75
*#N*/*Mol*. (*mean*)	Actin	198	534	542	576
	Tubulin	290	790	802	830
	Mitochondria	236	784	815	891
*#Molecules*	Actin	93076	10261	9759	8177
	Tubulin	25130	3379	3193	2718
	Mitochondria	112879	7552	6677	4925

## Discussions

A correlation-based single-molecule super-resolution localization microscopy (*corrSMLM*) technique is developed. It relies on the fact that fortunate molecules (molecules with long blinking periods) fluoresce for a longer time (than standard SMLM). These molecules are expected to appear on more than one frame and can be easily distinguished from random background noise that are not likely to repeat in subsequent frames. Thereby, eliminating these spurious events/false detections and retaining the genuine events (detection of fortunate molecules) result in better image quality (high resolution and SBR).

Single molecule experiments were performed in cellular systems, following specific biological protocols (cell culture, transfection and fixing). The cells were transfected to express photoactivable protein (Dendra2-Actin/Dendra2-Tubulin/mEos-Tom20) that are targeted to the specific organelle (such as, actin filaments, tubulin and mitochondrial network). Here, an event means signal photons i.e, emissions form photoactivatable proteins.

It may be noted that, non-specific events resembling single molecule blinks are most likely due to noise, background, and detector inefficiencies (read/thermal noise) in the specimen. When two or more consecutive frames are correlated, these noises are filtered out, thereby retaining only the genuine fortunate molecules. Hence, the resultant reconstructed images are free from the background. This is quite evident from Figs. 2–5. Another important aspect of *corrSMLM* is the correlation factor (taken between 0.7 to 0.9) for which the corresponding reconstructed super-resolved image appears to represent the target organelle and have shown efficient filtering of non-specific events (see, Fig. 3). This is encouraging since this emboldens the fact that there is a strong correlation of fortunate single molecule across successive recorded frames. To better understand the benefits of *corrSMLM*, a few regions were chosen, and intensity analysis along with the number of molecules representing fine sub-cellular structures (actin/tubulin/mitochondria) are carried out (see Figs. 5 and 6). The analysis indicates a significant reduction in background while retaining the single molecules that genuinely represent fine structure. This emboldens the advantage of detecting fortunate molecules in localization microscopy.

It may be noted that the proposed technique has its limitation too. In the case of high-density blinking, there is high chance for the overlapping of PSF both in the image and the subsequent images. This will make it difficult to identify fortunate molecules in the subsequent frames or the molecule that blink in more than 1 frame. So corrSMLM may not be a suitable method for high-density blinking. As far as 3D SMLM is concerned, corrSMLM can be used with some of the existing techniques that does not involve scanning. As the signals are captured from a same focal plane, so it is possible to correlate the PSF which repeats in the consecutive frames.

Finally, the proposed *corrSMLM* technique is compared with the standard SMLM. Analysis shows better localization, and an increase in the number of molecules that genuinely represent signal (as apparent from line intensity plots across actin filament, tubulin structures and mitochondrial network) (see, Fig. 6). In addition, the proposed technique is compared with ThunderSTORM, the details of which is discussed in Supplementary Note 10. The technique improves spatial resolution, localization accuracy (by temporally integrating the signal over all the images in which it appears), and eliminates false detections due to all possible sources of noise. Further analysis suggests the benefits of *corrSMLM* over SMLM in terms of mean localization precision, signal-to-background ratio, among others (see, Table 1). To quantify the effect of photobleaching during data acquisition, super-resolved images are reconstructed from single molecules collected at different time points of the experiment (early, intermediate, and end) as discussed in Supplementary Note 5. The results clearly indicate the negligible effect of photobleaching for the duration of data acquisition. In addition, bitwise XOR analysis (in the Frequency domain) suggests the preservation of high-frequency components corresponding to fine structures (actin filaments/tubulin/mitochondrial network). With all the benefits and non-requirement of additional hardware, *corrSMLM* is expected to advance single-molecule super-resolution microscopy across the spectrum of physical and biological sciences.

It is evident that the localization precision has improved for corrSMLM, however, the number of localizations has decreased. This necessitates large data acquisitions to reconstruct a super-resolved image for corrSMLM as compared to standard SMLM. While the technique is useful for standard data acquisition, the technique may not be suitable for high-density blinking. This increases the chance of overlapping PSF in the image and may have consequences in recognizing molecules in the subsequent frames. However, the localization density plays a critical role in determining resolution as high localization accuracy means little if there aren’t enough fluorophores to resolve a feature. Thus localization techniques must ensure enough detected fortunate molecules for high-resolution reconstructions. In this respect, FRC analysis for determining image resolution is a fairly acceptable metric. As per the Nyquist-Shannon theorem, the distance between two neighboring point emitters (here, single molecules) should be two times that of the desired resolution. So if the localization accuracy is *l*_*a*_, then the point emitters must be *l*_*a*_/2 for correct sampling. In any case, the resolution (r) cannot be better than *r*_*loc*_ = 2.3*σ*_*loc*_^59^.

In general, the determination of image resolution requires the knowledge of both localization precision and density of localization (DoL). DoL can be calculated by finding the number of localizations per unit area. The relative DoL is defined as, *ρ*_*DoL*_ = *DoL*_*SMLM*_/*DoL*_*corrSMLM*_ which is calculated to be, 1.558 for Dendra2-Actin sample. This indicates that DoL of *SMLM* is relatively larger than *corrSMLM*. The consequence of this is in determining resolution of the image^60^, ${R}^{2}={\sigma }^{2}+{{D}_{nn}}^{2}$, where, *D*_*nn*_ is the nearest neighbor distance. *D*_*nn*_ is calculated using inbuilt MATLAB scripts and is found to be 22.38*nm* and 29.15*nm* (Dendra2-Actin sample) for SMLM and *corrSMLM*, respectively. The corresponding median localization precision(*σ*) is calculated to be 33.9*nm* and 15.2*nm* for SMLM and *corrSMLM*. This gives an overall effective resolution of 40.62*nm* and 32.87*nm* for *SMLM* and *corrSMLM*, respectively. Though the nearest neighbor distance is slightly higher for *corrSMLM*, the effective resolution is better due to a two-fold improvement in the localization precision.

## Method

### Cell culture and transfection protocol

For *corrSMLM* study, we used three different specimens to super-resolve 3 distinct intra-cellular structures (actin filaments, tubulin and mitochondrial network). NIH3T3 cells with a passage number of 14 were thawn and supplemented with cell medium(89% Dulbecco’s Modified Eagle Medium, 10% Fatal Bovine Serum and 1% Penicillin-Streptomycin), and maintained at 37 ^*o*^*C* and 5% CO2. The cells were then cultured for two more passages to ascertain its healthy growth. Then the cells are seeded on a coverslip of size 22mm*22*mm* (No.0), with a count of 75,000 cells/ml and kept inside the incubator. 16 hours after seeding, cells are transiently transfected with Dendra2-Actin plasmid using Lipofectamine 3000 following the manufacturer’s protocol (Invitrogen, USA). We have used, 1*μ*g of plasmid DNA, 2.25 *μ*L of P3000 and 3 *μ*L lipofectamine. Cells were fixed after 60 hours of transfection using 3.7% paraformaldehyde and the coverslip was mounted on a glass slide using Fluorosave mount media. The same protocol is followed for Dendra2-Tubulin and mEos-Tom20.

### Data acquisition and image processing

In the first experiment, Dendra2-Actin plasmid DNA was used to transfect NIH3T3 cells. Dendra2 emits fluorescence signal at 507nm when it is excited using light of wavelength 490nm before photoconversion. This property of the dendra2 protein is used to indentify the transfected cells in the sample. The transfected cell image is recorded using Jenoptik ProgRes camera. Then EMCCD (Andor iXon life 897) camera was used to record the data/images. Before recording data, the camera was cooled to  −88 °C to reduce thermal noise. Data are recorded in frame transfer mode at an exposure time of 30*ms* and EM gain of 274. An estimated 49,020 images are recorded for reconstructing standard SMLM image as shown in Fig. 2. Subsequently, the images are corrected for drift and are correlated using Pearson Correlation Coefficient (*χ*) for 2, 3 and 4 successive frames (see, Supplementary Note 8). This is necessary to recognize fortunate molecules with long blinking time. The super-resolved image for *corrSMLM* is then reconstructed as shown in Fig. 2. In addition, another super-resolved image is reconstructed for 2-4 frames that combine all the single molecules detected for 2, 3, and 4 frames. χ is defined as,

1 $$\chi =\frac{{\sum }_{m}{\sum }_{n}({A}_{mn}-\bar{A})({B}_{mn}-\bar{B})}{\sqrt{({\sum }_{n}{\sum }_{m}{({A}_{mn}-\bar{A})}^{2})({\sum }_{n}{\sum }_{m}{({B}_{mn}-\bar{B})}^{2})}}$$

*A*_*mn*_ and *B*_*mn*_ are the two images of dimension *m* × *n* taken for correlation. $\bar{A}$ and $\bar{B}$ are the mean of *A*_*mn*_, *B*_*mn*_ respectively.

### Signal to background ratio

A rectangular region across the region of interest is considered. The average intensity and number of molecules in that region are estimated. Filament thickness is figured out using the Gaussian function fitted on the bar graph data, and the number of molecules on the filament and outside the filament are calculated. The SBR is calculated as the ratio of number of molecules on the filament and outside the filament.

### Entropy analysis

The entropy of an image is the statistical measurement of the randomness. It is evaluated using the expression,

2 $$E={\sum}_{i}{p}_{i}\times lo{g}_{2}{p}_{i}$$

where *p*_*i*_ is the histogram counts of the given image. The entropy study was performed on all three samples and it’s evident that the entropy of SMLM images is higher than *corrSMLM* images due to the presence of random background molecules.

### Fourier XOR analysis

The Fourier XOR analysis is a Boolean measure that compares the two similar datasets/images in Fourier domain. Consider, two images *f*_1_ and *f*_2_ for which the Fourier transforms are given by *F*_1_ and *F*_2_, respectively. The bitwise Fourier XOR between the images is given by,

3 $${F}_{1}\oplus {F}_{2}=\left\{\begin{array}{l}0,\,{F}_{1}(i,j)={F}_{2}(i,j)\,\,\forall \,i,j\quad \\ 1,\,{F}_{1}(i,j) \, \ne \, {F}_{2}(i,j)\,\,\forall \,i,j\quad \end{array}\right.$$

The process begins with taking the Fourier Transform (FFT) of both SMLM and corrSMLM images, followed by a bitwise XOR operation on the FFT images (FFT images are shown in Supplementary Note 7). In our case, this XOR operation helps nullifies common frequencies which are mostly low-frequency components (common for the FFT images) and highlights the high-frequency components that are present in corrSMLM images. It may be noted that, low frequencies are in the centre of FFT image and high frequencies away from the origin. Post Fourier XOR operation, most of the low-frequency components are nullified and retains only high-frequencies that appear as a hollow ring-like structure.

### Statistics and reproducibility

The statistical analysis like mean values, and ratios (SBR and localization precision) are calculated using Matlab code and mentioned in the discussion section. A sample size of *n* = 5 NIH3T3 cells, and 3 different plasmids (Dendra2-Actin, Dendra2-Tubulin and mEos-Tom20) were used. Also, additional experiments were performed to ensure reproducibility (see, Supplementary Note 9).

### Reporting summary

Further information on research design is available in the Nature Portfolio Reporting Summary linked to this article.

## Supplementary information

Description of Additional Supplementary Materials

Supplementary Notes 1 - 11

Supplementary Data

Supplementary Video 1

Supplementary Video 2

Supplementary Video 3

Supplementary Video 4

Supplementary Video 5

Supplementary Video 6

Supplementary Video 7

Reporting Summary

## Data Availability

Datasets generated and analyzed in this manuscript are given as, Supplementary Data 1. The raw data that support the findings of this study are available from the corresponding author upon request.

## Code availability

The entire code and related details can be found in GITHUB, and are available via Zenodo (https://zenodo.org/records/13968805)^61^. All scripts used to create the main and Supplementary Figs. are implemented in Matlab (version 2022b).
