# Supplementary material for: Fortunate molecules boost signal to background ratio and localization precision in correlation based single molecule localization microscopy
Source: Commun Biol. 2024 Dec 23;7:1693. doi: 10.1038/s42003-024-07153-x (PMC11666785; doi:10.1038/s42003-024-07153-x)
Supplement: Supplementary file 1 — Description of Additional Supplementary Materials [file 42003_2024_7153_MOESM1_ESM.pdf]

Description of Additional Supplementary Files

File name: Supplementary Data 1

Description: Numerical source data

File name: Supplementary Video 1

Description: Dendra2-Actin reconstructed image for SMLM and corrSMLM (c=0.7, 0.8, 0.9).

File name: Supplementary Video 2

Description: Comparative video of reconstructed images of Actin filaments (SMLM and corrSMLM).

File name: Supplementary Video 3

Description: Dendra2-Tubulin reconstructed image for SMLM and corrSMLM (c=0.7, 0.8, 0.9).

File name: Supplementary Video 4

Description: Comparative video of reconstructed images of Tubulin (SMLM and corrSMLM).

File name: Supplementary Video 5

Description: mEos-Tom20 reconstructed image for SMLM and corrSMLM (c=0.7, 0.8, 0.9)

File name: Supplementary Video 6

Description: Comparative video of reconstructed images of Mitochondrial Network (SMLM and corrSMLM).

File name: Supplementary Video 7

Description: Enlarge view of few chosen regions of mEos-Tom20 transfected cell
