# Supplementary material for: Fortunate molecules boost signal to background ratio and localization precision in correlation based single molecule localization microscopy
Source: Commun Biol. 2024 Dec 23;7:1693. doi: 10.1038/s42003-024-07153-x (PMC11666785; doi:10.1038/s42003-024-07153-x)
Supplement: Supplementary file 2 — Supplementary Notes 1 - 11 [file 42003_2024_7153_MOESM2_ESM.pdf]

Supplementary Material: Fortunate Molecules Boost Signal to Background Ratio and Localization Precision in Correlation based Single Molecule Localization Microscopy

Aravinth S[1], Francesca C. Zanacchi[3,4] and Partha P. Mondal[1,2]

[1] Department of Instrumentation and Applied Physics, Indian Institute of Science, Bangalore 560012, INDIA
[2] Centre for Cryogenic Technology, Indian Institute of Science, Bangalore 560012, INDIA
[3] Centre for Instrumental Sharing of the University of Pisa (CISUP), Pisa, ITALY
[4] Department of Physics, University of Pisa, Pisa, ITALY

Supplementary Notes 1-10

Supplementary Note 1: Data processing scheme for corrSMLM

Supplementary Note 2: corrSMLM reconstructed images of Dendra2-Actin transfected cells for χ=0.8 & 0.9.

Supplementary Note 3: corrSMLM reconstructed images for mEos-Tom20 transfected cells.

Supplementary Note 4: Localization precision analysis for Dendra2-Actin, Dendra2-Tubulin and mEos-Tom20 transfected cells.

Supplementary Note 5: Statistical analysis for temporal data collection.

Supplementary Note 6: Fourier Ring Correlation Analysis for Dendra2-Actin, Dendra2-Tubulin and mEos-Tom20.

Supplementary Note 7: Fourier Analysis of super-resolved SMLM and corrSMLM images

Supplementary Note 8: Lateral drift-correction for *corrSMLM.*

Supplementary Note 9: Additional Data for Dendra2-Actin Transfected Cells

Supplementary Note 10: Comparative Study of corrSMLM & ThunderSTORM

Supplementary Note 11: Histogram of the T_{ON} of the fluorophores at varying exposure time.

Supplementary Videos:

Supplementary Video 1: Dendra2-Actin reconstructed image for SMLM and corrSMLM (χ=0.7, 0.8, 0.9).

Supplementary Video 2: Comparative video of reconstructed images of Actin filaments (SMLM and corrSMLM).

Supplementary Video 3: Dendra2-Tubulin reconstructed image for SMLM and corrSMLM (χ=0.7, 0.8, 0.9).

Supplementary Video 4: Comparative video of reconstructed images of Tubulin (SMLM and corrSMLM).

Supplementary Video 5: mEos-Tom20 reconstructed image for SMLM and corrSMLM (χ=0.7, 0.8, 0.9).

Supplementary Video 6: Comparative video of reconstructed images of Mitochondrial Network (SMLM and corrSMLM).

Supplementary Video 7: Enlarge view of few chosen regions of mEos-Tom20 transfected cell.

Supplementary Note 1. Data processing scheme for corrSMLM

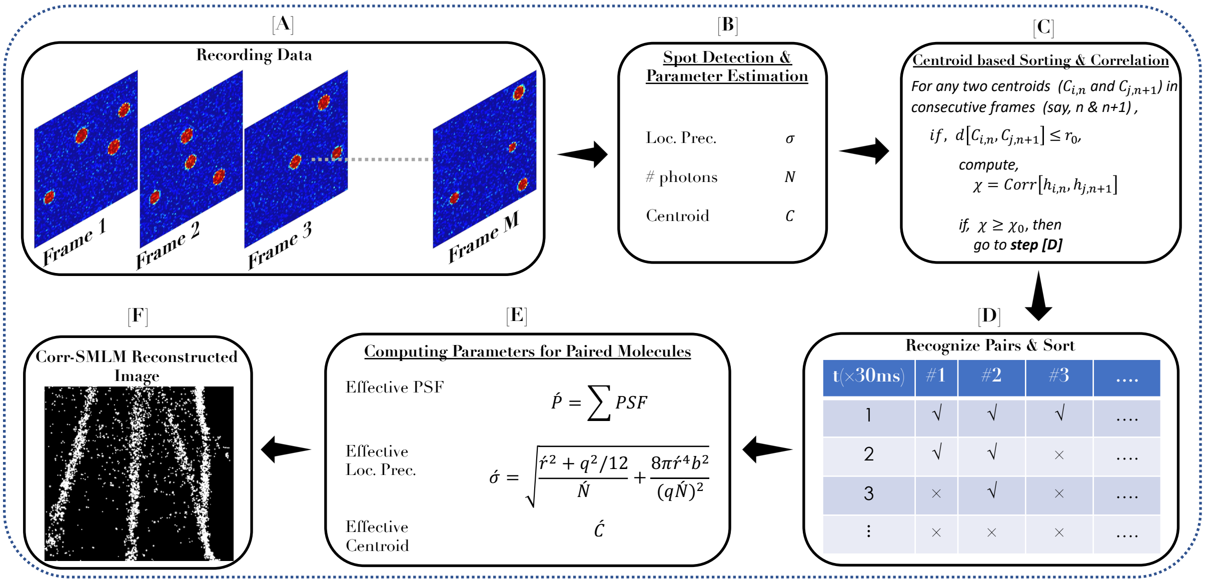

t(×30ms)	#1	#2	#3	....
1	√	√	√	....
2	√	√	×	....
3	×	√	×	....
⋮	×	×	×	....

Fig. S1. corrSMLM Workflow. The process involved in corrSMLM begins with recording data / images followed by, spot-detection, correlation, pair recognition, parameter estimation (#photons, localization precision and location) and image reconstruction.

Single molecule blinking events are recorded using the EMCCD camera with a frame rate of 33Hz in TIFF file format. The recorded data are processed using MATLAB program. The process begins with removing the background, followed by thresholding to identify the single molecule spots. Then the Centroid $C_{i,n}$ (Centroid of ith molecule in n^{th} frame), maximum intensity ($a_{i,n}$), and radius ($r_{i,n}$), (distance from the centroid to a point where intensity decreases to $1/e^2$ of maximum intensity) of each spot are estimated using the nonlinear least square fitting method. 2D Gaussian function is taken as a fit function. To identify fortunate molecules from SMLM-dataset, multiple localizations of a single molecule needs to be identified. This is achieved by calculating centroid distance in consecutive frames and the corresponding correlation factor. corrSMLM finds the Euclidean distance between the centroids of each molecule in consecutive frames n and n+1. Suppose the Euclidean distance is less than the cutoff (which is the $r_0 = \lambda/2NA$ value of the respective molecule in frame n), then two PSFs are generated using ($C_{i,n}$, $a_{i,n}$, $r_{i,n}$) of the molecule and correlation factor χ between the generated PSF are calculated using equation 1 (see, main text). When the correlation factor is greater than pre-determined cutoff (say, 0.7), then the two molecular localizations are assumed to belong to a single molecule, and the pair of localizations are noted (see, Fig. S1D, Table). The same analysis is carried out for all the consecutive frames. Using the localization pair, molecules are sorted based on their blinking time from 60ms to 120ms. After sorting out the molecules based on their blinking time, the effective PSF ($\acute{P}$) is calculated by merging their PSF in consecutive frames into a single PSF using matrix addition. The effective centroid ($\acute{C}$), effective radius ($\acute{r}$) and total number of photons ($\acute{N}$) of a single molecule repeated in multiple frames are calculated by fitting 2D gaussian function on the effective PSF. Using $\acute{r}$ and $\acute{N}$ the effective localization precision ($\acute{\sigma}$) is calculated. The final super-resolution image is reconstructed using $\acute{C}$ and $\acute{\sigma}$ of all the detected molecules.

Supplementary Note 2. corrSMLM reconstructed images of Dendra2-Actin transfected cells for χ=0.8 & 0.9.

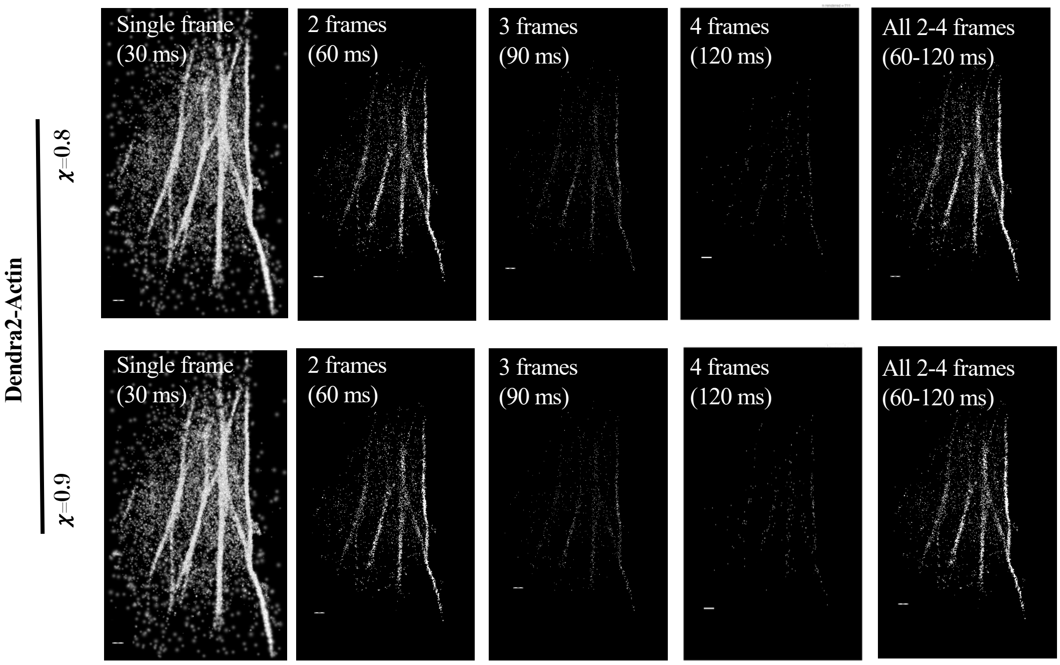

Fig. S2-1. corrSMLM reconstructed image of Actin Filaments. Reconstructed images of Actin filaments for varying blinking times (30 ms to 120 ms) and two different correlation factors, χ=0.8 and 0.9

A standard super-resolved image require somewhere between 5000-15000 images to accurately represent structures, but high values of correlation factor limits the number of molecules. Hence, an alternate strategy could be to collect large number of frames to compensate for the number, but with an increased risk of system drift (see, Supplementary Note 8). So, a balance is essential to reconstruct a super-resolved map using corrSMLM technique.

Fig. S2-1 shows corrSMLM images of Dendra2-Actin for a correlation factor of, χ = 0.8 and 0.9. Alongside SMLM reconstructed images are also shown (for molecules that appear in a single frame. Last column show images reconstructed using all the molecules appearing in 2-4 frames). It is evident that, image quality improves for corrSMLM (all 2-4 frames), but the number of molecules decrease as the blinking time of the molecules increases. This necessitates longer acquisition times. This is also the reason why the analysis is limited to 4 frames.

Supplementary Note 3. corrSMLM reconstructed images for mEos-Tom20 transfected cells.

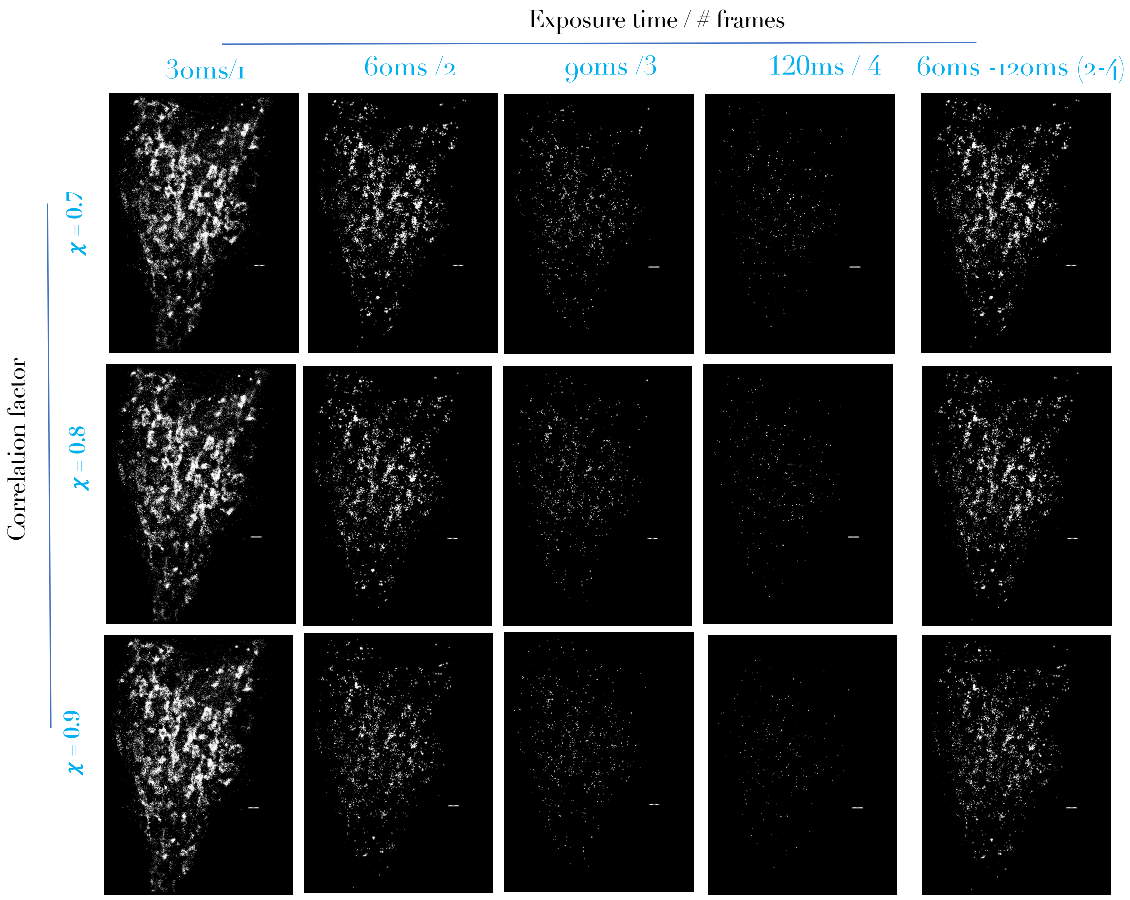

Fig. S3-1. corrSMLM reconstructed images of mitochondrial network: The illustration shows the corrSMLM super-resolved images at varying exposure times (30-120 ms) and for three different correlation values, χ=0.7, 0.8, 0.9.

To visualize the mitochondrial network with single molecule precision, NIH3T3 cell were transfected and imaged. The reconstruction were obtained for different correlation factors (χ=0.7, 0.8, 0.9) and for different exposure times as shown in Fig. S3-1. Subsequently, the final image is obtained by superimposing the data obtained for fortunate molecules with exposure times, 30-120 ms or equivalently molecules that appear in more than 1 frame i.e, 2-4 frames. It is evident that, the final super-resolved image displays a well-structured and discrete representation of mitochondrial network. On the other hand large correlation factor (here, χ=0.9) shows slightly better signal to background ratio.

This suggests that better resolved images are obtained at high correlation factor and for images reconstructed using fortunate molecules. This is due to the fact that spots appearing on more than 1 frame most likely represent single molecules with large blinking period, whereas single frame spots (< 30 ms) may have a significant fraction due to random noise. Overall, the technique facilitates precision analysis of the target protein (here, Tom20) in the respective organelle.

Supplementary Note 4. Localization precision analysis for Dendra2-Actin, Dendra2-Tubulin and mEos-Tom20 transfected cells

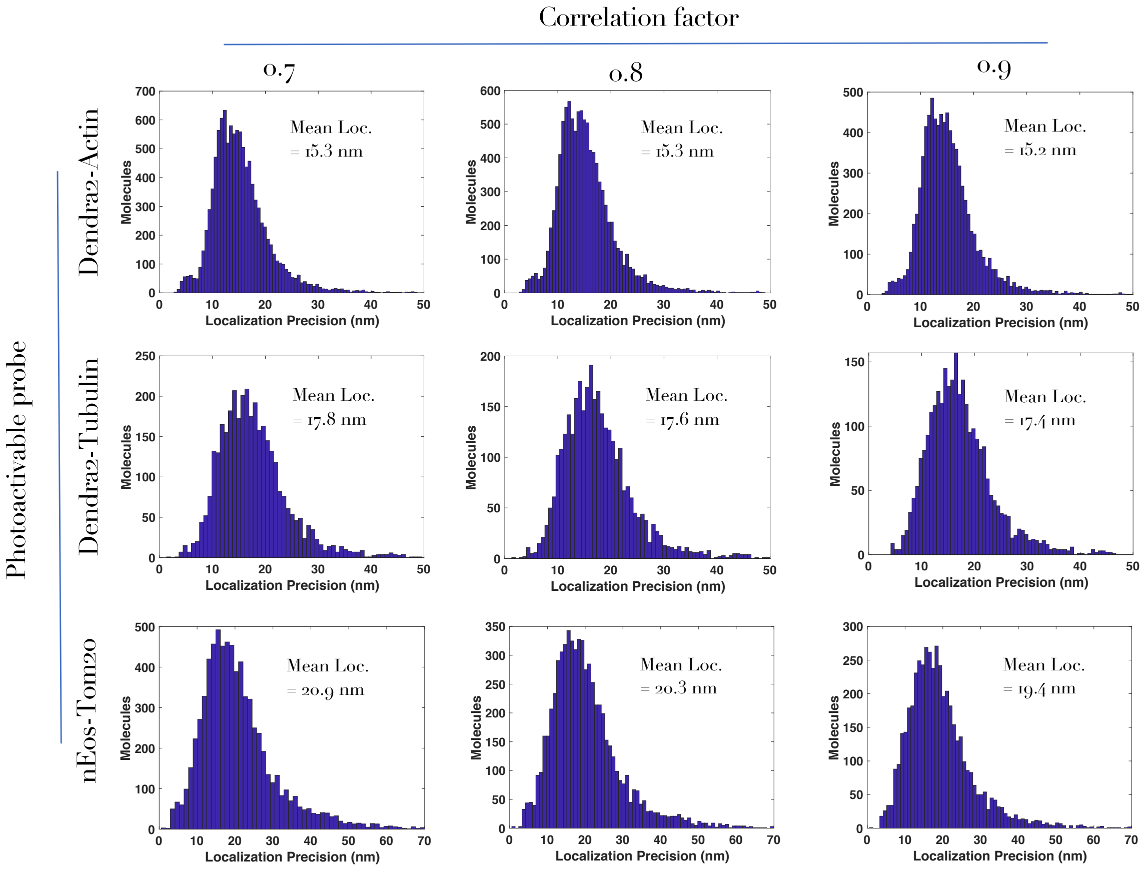

Fig. S4-1. Localization Precision Analysis. The characterization of localization precision for three different organelles (Actin, Tubulin and Mitochondria labelled using Dendra2 and mEoS photoactivable protein) at different correlation factors.

Localization precision is essential for determining the number of photons detected per molecule and directly influences the achievable spatial resolution. Fig. S4-1 shows the localization precision for the single molecules, Dendra2-Actin, Dendra2-Tubulin and mEos-Tom20, that labels Actin, Tubulin and Mitochondria, respectively. The histogram plots correspond to correlation factors (χ=0.7, 0.8, 0.9). It is evident that, average localization precision for Dendra2-Actin, Dendra2-Tubulin and mEos-Tom20 are 13.5nm, 15.4 nm, and 18nm, respectively. This is ~2 times less compared to a typical SMLM. This demonstrates the ability of the proposed corrSMLM technique for better localization of single molecules, ultimately resulting in better resolution. In addition, significant change is not noted in localization precision with correlation factor, suggesting that a factor of 0.7 is acceptable for corrSMLM.

Supplementary Note 5. Statistical analysis for temporal data collection

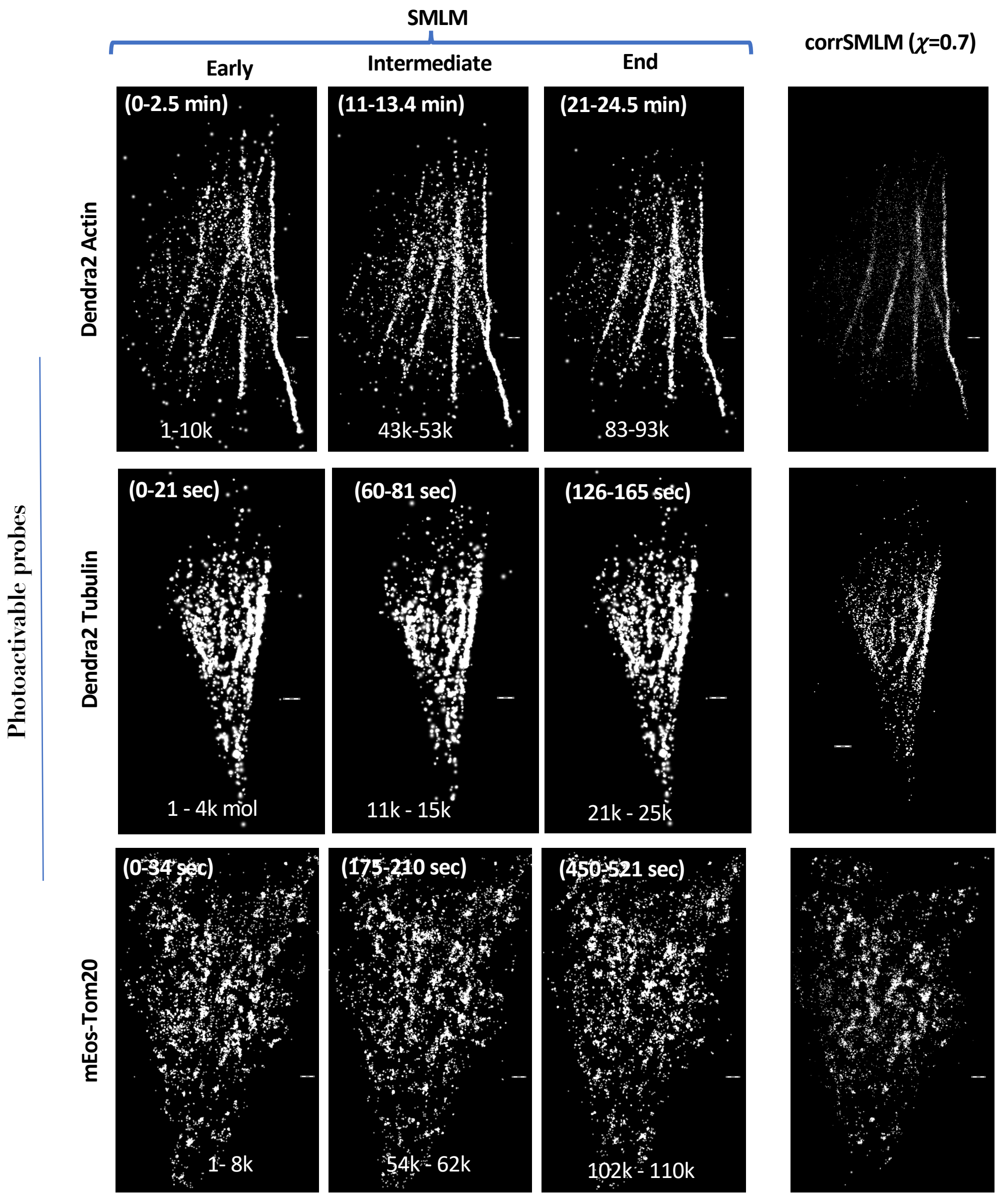

Fig. S5-1. Comparison of SMLM and *corrSMLM* reconstructed images at varying temporal window. Reconstructed images are compared for molecules detected early, intermediate and end stages of data acquisition.

The effect of photobleaching at different stages of data acquisition is crucial for quality imaging. To understand, we carried out analysis at different stages / time-window of data acquisition. This will help assess the impact of photobleaching on image quality. Three different time windows are chosen, early, intermediate, and end, as shown in Fig. S5-1. The corresponding time-window is mentioned for the respective SMLM images. This removes the bias of data collection as far as time of acquisition is concerned. We did not observe visible effect of photobleaching on the reconstructed images (see, Fig. S5-1). Alongside, corrSMLM images are also displayed that appear crisp and the background noise is substantially reduced as compared to standard SMLM. This proves the effectiveness of the proposed correlation-based technique for high-quality super-resolution imaging.

Supplementary Note 6. Fourier Ring Correlation Analysis for Dendra2-Actin, Dendra2-Tubulin and mEos-Tom20.

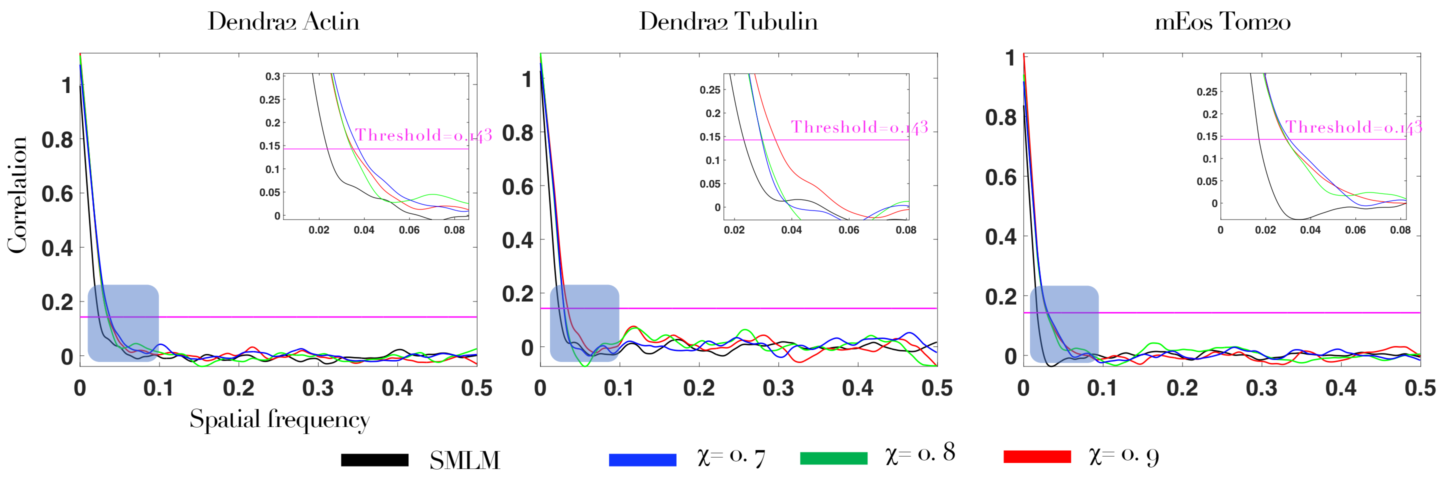

Fig. S6-1. FRC analysis. The analysis is carried out for three different cell samples, and a comparison is drawn between corrSMLM (χ=0.7, χ=0.8 and χ=0.9) and standard SMLM.

Fourier Ring Correlation (FRC) analysis is carried out for SMLM and corrSMLM(χ=0.7, χ=0.8 and χ=0.9) as shown in Fig. S6-1. FRC approximately determines the resolution of super-resolution images [1]. First, the reconstructed image is split into two sub-images (A and B) by distributing the molecules alternatively in each image. The sub-image A takes 1st, 3rd ,5th , … molecules and B takes 2nd, 4th, 6th, … molecules. Subsequently, the sub-images are Fourier transformed, and the correlation factor is calculated along the concentric rings for all possible radius. The correlation factor vs spatial frequency graph is plotted after smoothing the noisy data for each case. Resolution is calculated by finding the inverse of spatial frequency at which the correlation factor falls below the threshold (1/7). It is evident that corrSMLM produces better resolved images as compared to standard SMLM images for all three samples. This is evident form the sub-plot (inset in Fig. S6-1) in each figure. The corresponding values are tabulated in Table S6-T.

	Resolution (nm)			
Sample	SMLM	corrSMLM χ=0.7	corrSMLM χ=0.8	corrSMLM χ=0.9
Dendra2 Actin	169	112	116	107
Dendra2 Tubulin	171	116	134	132
mEos Tom20	232	137	136	131

Table S6-T. Comparison of resolution for corrSMLM (χ=0.7, 0.8, 0.9) and SMLM.

Supplementary Note 7. Fourier Analysis of super-resolved SMLM and *corrSMLM* images

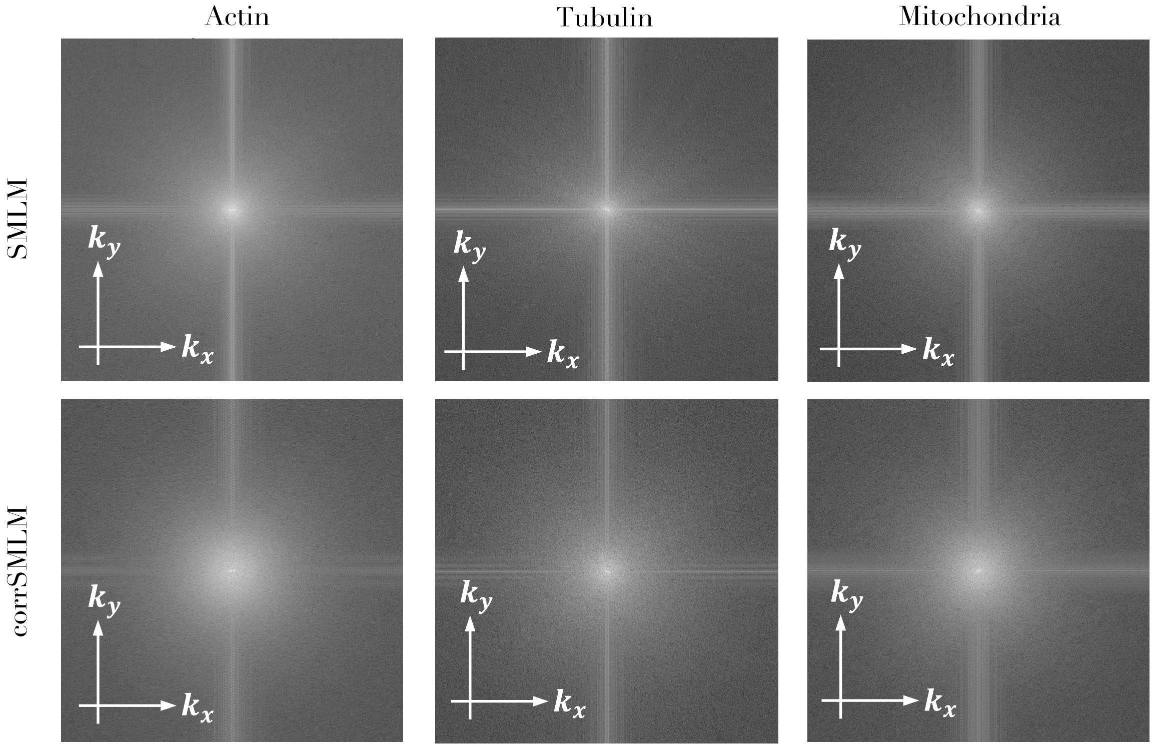

Fig. S7-1. Fourier Analysis : Fast Fourier Transform(FFT) of SMLM and corrSMLM images of transfected NIH3T3 cells (Dendra2-Actin, Dendra2-Tubulin and mEos-Tom20).

In single molecule localization microscopy, spatial frequency components of the super-resolved image plays critical role, specifically for determining image resolution. Fig. S7-1 shows FFT analysis in Fourier domain for 3 different specimens, comparing SMLM and corrSMLM. The analysis is used for XOR analysis that compares spatial frequencies present in both the images. The first step is to find the spatial frequency present in SMLM and corrSMLM reconstructed images using FFT. The amplitude spectrum of FFT images for SMLM and corrSMLM are shown in first and second row respectively. Here, k_x and k_y are the spatial frequencies along horizontal (x) and vertical (y) axis. These amplitude spectrum are further subjected to pixel-wise XOR operation. It is evident that, corrSMLM has a large share of high frequencies as compared to standard SMLM. This strongly indicates strong support for edges and fine features in the super-resolved image.

Supplementary Note 8. Lateral drift correction for corrSMLM

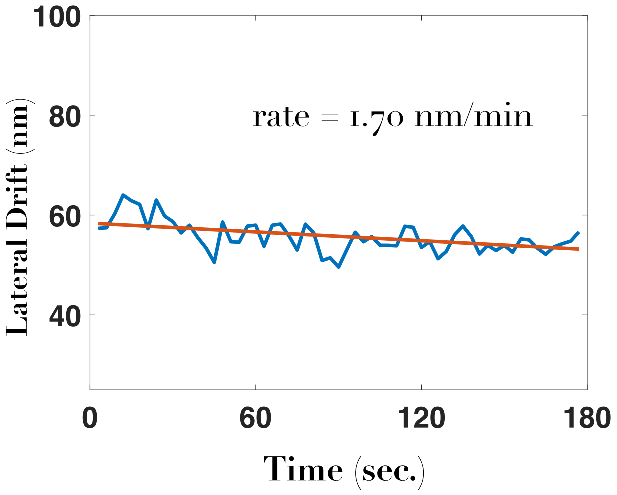

Fig. S8-1. Lateral XY drift correction : The stage drift of the sample stage in the lateral XY axes during data acquisition for corrSMLM.

Single molecule localization microscopy requires calibration for stage drift correction. The system drift correction was calculated using nano-beads.

The drift correction was carried out using fluorescent bead of size 170nm (Invitrogen. Excitation – 505nm. Emission - 515nm). 2uL of nanobead solution is mixed uniformly with 2 mL of 3% agarose gel at 42°C. This mixture was poured on 35 mm live imaging dish (Mat Tek, Ashland, USA) and allows it to solidify. A single isolated fluorescent bead from the live imaging dish is identified for imaging. The selected bead was imaged with an exposure time of 60ms using EMCCD camera. For every 3 seconds one frame was recorded, this was continued up to the actual data collection time (~ 180 seconds) for biological samples, so a total of 60 frames are recorded. Then the centroid of the bead in each frame was computed. A reference point (origin) was chosen and the distance between the reference point and each centroid is calculated and plotted with respect to time. This gives a drift rate of 1.70 nm/min as shown in Fig. S8-1. The drift was taken into account for analysing single molecule data.

Supplementary Note 9. Additional Data for Dendra2-Actin Transfected Cells

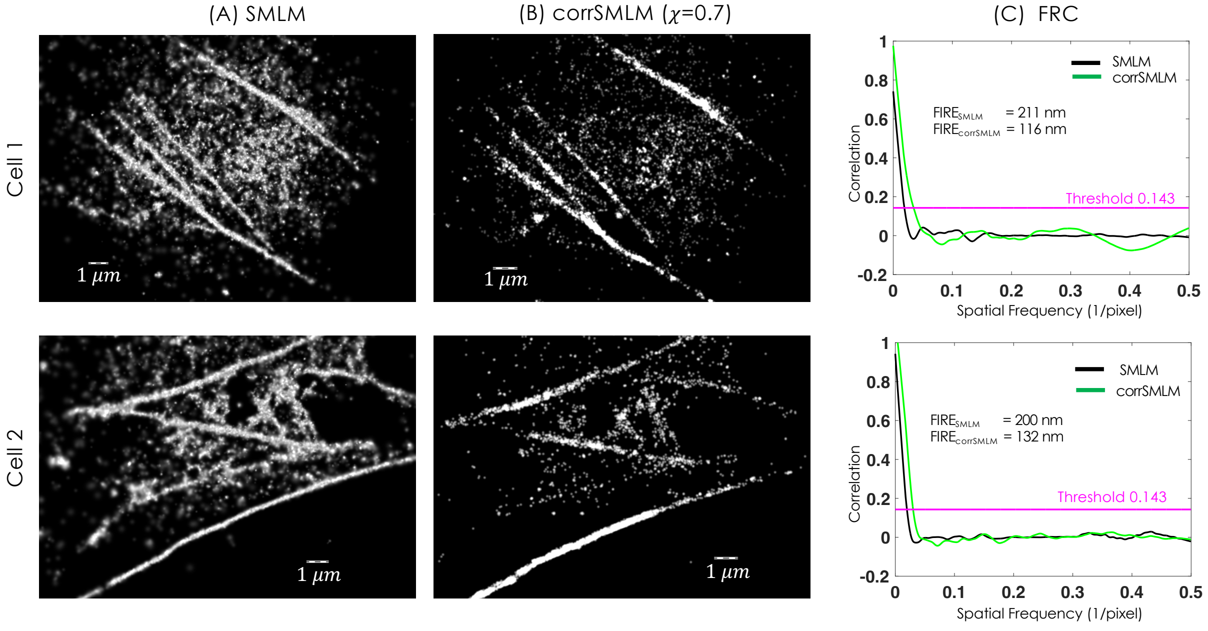

Fig. S9-1. Reconstructed image and FRC analysis: (A, B) corrSMLM and SMLM reconstructed images for two additional cells (cell1 and cell2. (C) FRC analysis and resolution comparison for SMLM and corrSMLM. Scale bar is 1 μm.

Additional experiments are carried out on Dendra2-Actin transfected cells, and the data (image stacks) are obtained for further analysis. Both corrSMLM and standard SMLM techniques are validated. Fig. S9-1(A,B) shows the reconstructed images for both the cells. The corrSMLM reconstructed images are obtained for a correlation factor of, χ=0.7. It is quite evident that corrSMLM technique is able to filter out the random noise and retain genuine structures (here Actin bundles). In addition, FRC analysis is carried out to determine image resolution as shown in Fig. S9-1C. The corresponding FIRE values are found to be 116 nm and 132 nm for corrSMLM, whereas it is 211 nm and 200 nm for standard SMLM. This suggests that the resolution of corrSMLM is approximately 2 times better than SMLM. Overall, corrSMLM can reconstruct images with high resolution and reduced background noise.

Supplementary Note 10. Comparative Study of corrSMLM & ThunderSTORM

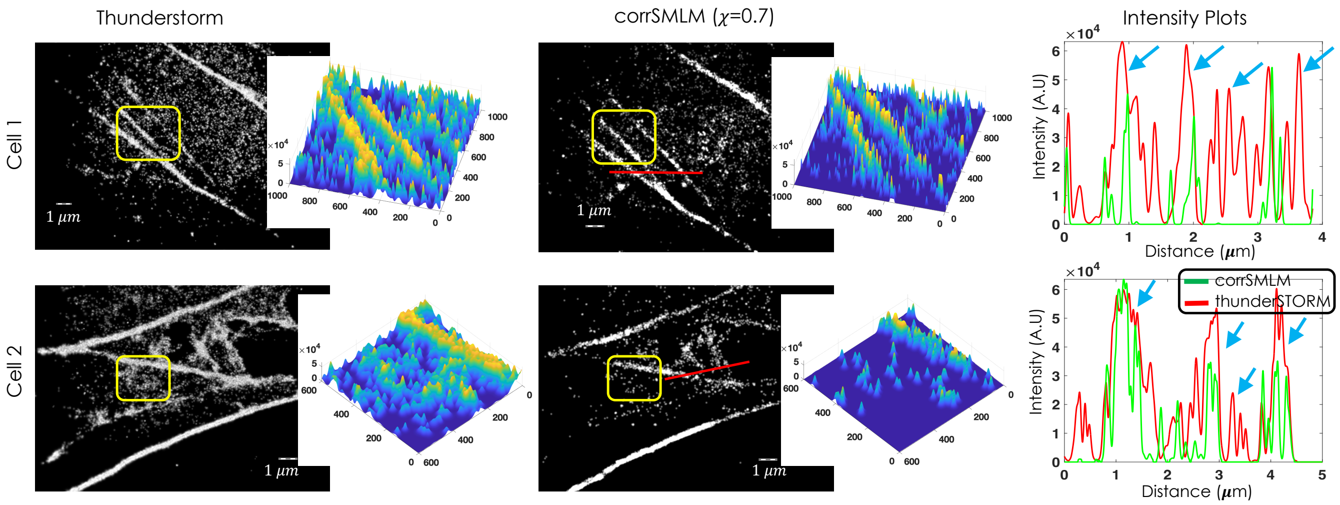

Fig. S10-1. Comparative Study: Reconstructed images of Dendra2-Actin using corrSMLM and Thunderstorm. Alongside, 3D surface plots and intensity plots (along red line) are also shown. The cells (cell1 and cell2) were taken from two independent experiments. Scale bar is 1 μm.

The proposed technique (corrSMLM) is compared with the well-known ThunderSTORM technique. Two cells (here, cell1 and cell2) were chosen from two independent transfected specimens. The raw data is processed using ThunderSTORM ImageJ plugin[1] and *corrSMLM,* for which the reconstructed images are shown in Fig. S9-1.

For the ThunderSTORM technique, the images are filtered using wavelet filter and molecules are approximately localized using local maximum method [2]. The single molecule PSF is considered as Gaussian and it is fitted with least square method for sub-pixel localization. Initial fitting radius and sigma values are 5 and 2 pixels respectively. After localizing molecules from all the frames thresholding is carried out on localization precision, number of photons and sigma values to have legitimate molecules. The molecules which repeats in multiple frames are merged using merging option in ThunderSTORM. The maximum number of frames for which molecules should be merged is considered as unlimited (by selecting zero). The maximum merging radius is selected as 200 nm. The super-resolution image is reconstructed after filtering and merging process and the image is compared with corrSMLM.

Visually, it is apparent that corrSMLM reduce background and random noise (see, Fig. S9-1). To exemplify, enlarged sections are also shown which supports the noise and background suppression ability of corrSMLM. To quantize the performance, we used line intensity plots (along red line). It is evident that the molecules representing actin structures and fine features (shown by blue arrows) are better preserved by corrSMLM. Overall, corrSMLM performs better than ThunderSTORM as far as background is concerned.

Supplementary Note 11. Histogram of the T_{ON} of the fluorophores at varying exposure time

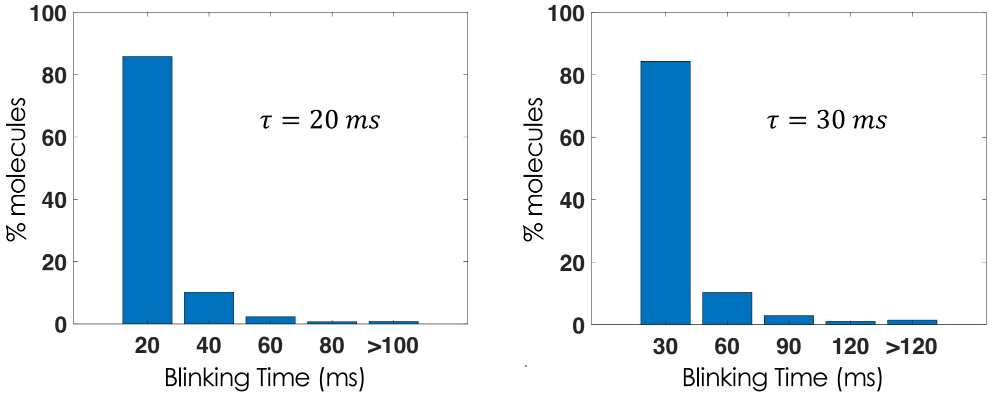

Exposure time	Percentage of molecules appearing in				
	1 frame	2 frames	3 frames	4 frames	⩾5 frames
20ms	85.8	10.2	2.3	0.7	0.8
30ms	84.3	10.28	2.84	1.04	1.46

Fig. S11-1. Histogram of the T_{on} of fluorophore at exposure times, τ=20 ms and 30 ms. The table compares percentage molecules at standard exposure time (30 ms) and at a relatively smaller exposure time (20 ms).

To understand the effect of exposure time on the blinking time (T_{on}) of the fluorophore, we carried out imaging at a lower exposure time (see, Fig. 11-1). Specifically, the study is performed at an exposure time, of t=20 ms to determine the percentage of single molecules that blink appear in more than 1 frame. The same is shown in Fig. S11-1. It is quite evident that a large fraction of molecules does not repeat i.e., do not appear in more than 1 frame. This is because molecules that blink for a longer time are less in proportion and a substantial contribution comes from false detections (arising due to electronics, fluorescent background noise, false detections, etc). A comparison of standard exposure time (30 ms) versus lower exposure time (20 ms) is tabulated in Fig. S11-1. Although the percentage of single molecules appearing in subsequent frames (2 or more) changes for 20 ms exposure, a slightly larger percentage is noted for 30 ms exposure. Specifically, 10.28%, 2.84%, 1.04%, 1.46% of molecules appear in 2, 3 , 4 and 5 frames for an exposure time of 20 ms. This strengthens the fact that the T_{on} of the fluorescent molecule is larger than 20 ms, and supports the choice of 2 frames for the standard 30 ms exposure time.

References

1. Nieuwenhuizen, R., Lidke, K., Bates, M. et al. Measuring image resolution in optical nanoscopy. Nat Methods 10, 557–562 (2013).
2. M. Ovesný, P. Křížek, J. Borkovec, Z. Švindrych, G. M. Hagen. ThunderSTORM: a comprehensive ImageJ plugin for PALM and STORM data analysis and super-resolution imaging. Bioinformatics 30(16):2389-2390, 2014.
